# Supplementary material for: Oxidative Stress-Mediated Repression of Virulence Gene Transcription and Biofilm Formation as Antibacterial Action of Cinnamomum burmannii Essential Oil on Staphylococcus aureus
Source: Int J Mol Sci. 2024 Mar 6;25(5):3078. doi: 10.3390/ijms25053078 (PMC10932263; doi:10.3390/ijms25053078)
Supplement: Supplementary file 1 [file ijms-25-03078-s001.zip › ijms-2853794-supplementary.pdf]

Table S1. Primer sequences for quantitative real-time-PCR (qRT-PCR) used in this study

Gene	Forward primer sequence	Reverse primer sequence
sigB	5′-AAGTGATTCGTAAGGACGTCT-3′	5′-TCGATAACTATAACCAAAGCCT-3′
agrA	5′-TGATAATCCTTATGAGGTGCTT-3′	5′-CACTGTGACTCGTAACGAAAA-3′
sarA	5′-TCCCTTCAAAACCAAACGAA-3′	5′-AATTCAGGACATGCACCACA-3′
icaA	5′-GGAAGTTCTGATAATACTGCTG-3′	5′-GATGCTTGTTTGATTCCCTC-3′
cidA	5′-AGCGTAATTTCGGAAGCAACATCCA-3′	5′-CCCTTAGCCGGCAGTATTGTTGGTC-3′
rsbU	5′-AGCGTTTGAGGAAATTGGTGT-3′	5′-CCTCTACATCTCGTGCCTCTG-3′
16S rRNA	5′-GGGACCCGCACAAGCGGTGG-3′	5′-GGGTTGCGCTCGTTGCGGGA-3′

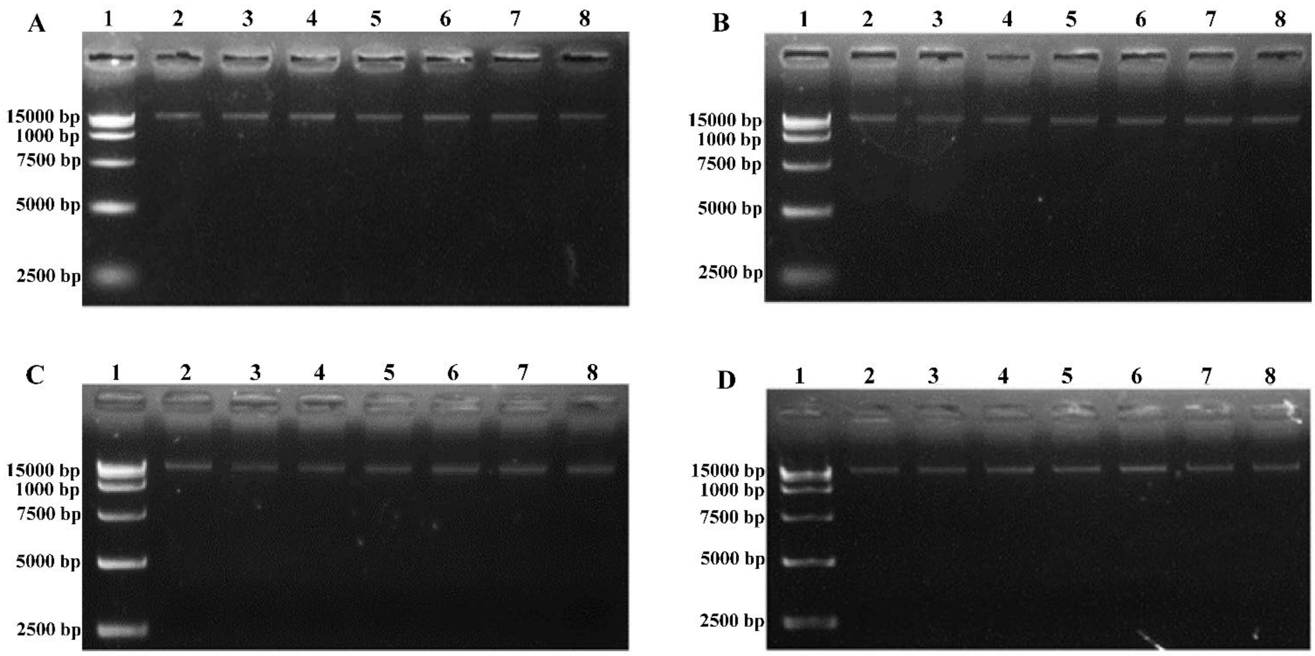

Figure S1. Effect of CBLEO on genome DNA of *S. aureus* cells by gel electrophoresis assay. **(A)** After 0.5 h incubation with different levels of CBLEO; **(B)** After 1 h incubation with different levels of CBLEO; **(C)** After 3 h incubation with different levels of CBLEO; **(D)** After 5 h incubation with different levels of CBLEO. Lane 1 signified DNA Marker; Lane 2 signified genome DNA of *S. aureus* treated by PBS (as the control); Lanes 3-8 signified genome DNA of *S. aureus* treated with 1/2×MIC, 1×MIC, 2×MIC, 4×MIC, 8×MIC and 16×MIC of CBLEO, respectively.
